# Immune gene expression and functional networks in distinct lupus nephritis classes

**DOI:** 10.1136/lupus-2021-000615

**Published:** 2022-01-24

**Authors:** Alyssa C Gilmore, Hannah R Wilson, Thomas D Cairns, Marina Botto, Liz Lightstone, Ian N Bruce, Herbert Terence Cook, Matthew Caleb Pickering, the MASTERPLANS Consortium

**Affiliations:** 1 Department of Immunology and Inflammation, Imperial College London, London, UK; 2 Imperial Lupus Centre, Imperial College Healthcare NHS Trust, London, UK; 3 Centre for Epidemiology Versus Arthritis, Faculty of Biology, Medicine and Health, The University of Manchester and NIHR Manchester Biomedical Research Centre, Manchester University Hospitals NHS Foundation Trust, Manchester Academic Health Science Centre, Manchester, United Kingdom, The University of Manchester, Manchester, UK

**Keywords:** lupus nephritis, lupus erythematosus, systemic, inflammation

## Abstract

**Objective:**

To explore the utility of the NanoString platform in elucidating kidney immune transcripts for class III, IV and V lupus nephritis (LN) using a retrospective cohort of formalin-fixed paraffin-embedded (FFPE) kidney biopsy tissue.

**Methods:**

Immune gene transcript analysis was performed using the NanoString nCounter platform on RNA from LN (n=55), thin basement membrane (TBM) disease (n=14) and membranous nephropathy (MN) (n=9) FFPE kidney biopsy tissue. LN samples consisted of single class III (n=11), IV (n=23) and V (n=21) biopsies with no mixed lesions. Differential gene expression was performed with NanoString nSolver, with visualisations of volcano plots and heatmaps generated in R. Significant transcripts were interrogated to identify functional networks using STRING and Gene ontogeny terms.

**Results:**

In comparison to TBM, we identified 52 significantly differentially expressed genes common to all three LN classes. Pathway analysis showed enrichment for type I interferon (IFN) signalling, complement and MHC II pathways, with most showing the highest expression in class IV LN. Our class IV LN biopsies also showed significant upregulation of NF-κB signalling and immunological enrichment in comparison to class V LN biopsies. Transcripts from the type I IFN pathway distinguished class V LN from MN.

**Conclusion:**

Our whole kidney section transcriptomic analysis provided insights into the molecular profile of class III, IV and V LN. The data highlighted important pathways common to all three classes and pathways enriched in our class IV LN biopsies. The ability to reveal molecular pathways in LN using FFPE whole biopsy sections could have clinical utility in treatment selection for LN.

**Key messages**

**What is already known about this subject?**

- The existing lupus nephritis (LN) histological classification system is used to direct treatment but a limitation is the lack of any information on the molecular pathogenesis underlying glomerular inflammation.

**What does this study add?**

- In comparison to controls (thin basement membrane disease kidney biopsies), we identified 52 significantly differentially expressed genes common across class III, IV and V LN biopsies using formalin-fixed paraffin-embedded (FFPE) whole kidney biopsy sections.
- Pathway analysis showed enrichment for type I interferon (IFN) signalling, complement and MHC II pathways with most showing the highest expression in class IV LN.
- Transcripts from the type I IFN pathway distinguished class V LN from membranous nephropathy.

**How might this impact on clinical practice or future developments?**

- The ability to reveal molecular pathways in LN using FFPE whole biopsy sections could have clinical utility in selecting patients for emerging targeted therapy.

## Introduction

Lupus nephritis (LN) is an important manifestation of systemic lupus erythematosus (SLE) and develops in approximately 40% of patients.^1^ Its prevalence is influenced by age (higher in childhood-onset SLE) and ethnicity (higher in non-Caucasian patients) and is inversely associated with socioeconomic status.^2^ The kidney biopsy is an important part of the management of LN^3 4^ and significant efforts have been made to relate the changes seen in the biopsy to pathogenesis, treatment selection and prognosis.^1^ Critical to this was the development of a LN classification system that incorporated standardised definitions of biopsy lesions to reduce inter-reporting variability.

The International Society of Nephrology/Renal Pathology Society (ISN/RPS) 2003 consensus classification of LN^5^ described six classes: minimal mesangial LN (class I); mesangial proliferative LN (class II); focal LN (class III); diffuse LN which could be further divided into diffuse segmental (class IV-S) and diffuse global (class IV-G) proliferative LN; membranous LN (class V) and advanced sclerosing LN (class VI). Active (A, eg, endocapillary hypercellularity, fibrinoid necrosis, cellular and fibrocellular crescents) and chronic (C, eg, glomerular sclerosis, fibrous crescents) glomerular lesions were also defined and denoted when describing classes III and IV. For example, class IV-G (A) described diffuse global proliferative LN with active lesions. An update to this classification has been proposed.^6^ Recommendations included the elimination of the class IV-S and IV-G subdivisions since these do not influence outcome. In a meta-analysis, the occurrence of either end-stage kidney disease or doubling of serum creatinine did not differ between IV-S and IV-G classes.^7^ It was also proposed to replace the active and chronic descriptors with activity and chronicity indices derived from the NIH lupus activity and scoring system,^8^ thereby enabling a quantitative assessment of activity (a score of 0 to 24) and chronicity (a score of 0 to 12).

The LN class is critical for informing management.^3^ Immunosuppression is recommended in active class III and IV LN but not in class II LN. The decision to instigate immunosuppression in class V LN is influenced by the degree of proteinuria and its response to renin-angiotensin-aldosterone-blockade. Failure to control proteinuria 12 months after treatment is associated with progression to chronic kidney disease^9–11^ so the management goal is to reduce proteinuria aiming for a complete clinical response (<0.5–0.7 g per 24 hours).

The LN class alone is less robust in informing outcome because risk factors for developing chronic kidney disease include clinical features and because they include histological features not captured by the LN class definitions. These include the degree of interstitial fibrosis and tubular atrophy (IFTA) and specific glomerular lesions such as fibrinoid necrosis and fibrous crescents.^12^ Quantification of selected and well-defined individual kidney lesions can inform prognosis. This is evident in IgA nephropathy where scoring of mesangial cellularity, endocapillary hypercellularity, segmental sclerosis, tubular atrophy, interstitial fibrosis and crescents to form the MEST-C score has been shown to aid risk prediction.^13–15^ The proposed revisions to the ISN/RPS 2003 consensus classification of LN might address these shortfalls^6^ but is still predicated on the use of morphology. Consequently, there is a need to study the molecular pathways within LN biopsies and understand how these relate to LN classes^16 17^ and treatment outcome.^18 19^ In terms of clinical utility, the transcriptomic analysis of kidney biopsies is most advanced in the diagnosis of antibody-mediated kidney rejection where an increase in expression of gene transcripts in biopsy tissue has, pending validation, been added to validated morphological criteria.^20^ To determine the utility of using transcriptomic data to improve the clinical utility of the kidney biopsy in SLE, we used NanoString technology to study the expression of 750 immune and inflammation-related transcripts in formalin-fixed paraffin-embedded (FFPE) sections from 55 LN biopsies, 14 biopsies with thin basement membrane (TBM) disease and 9 biopsies with membranous nephropathy (MN).

## Methods

Samples: FFPE kidney biopsy tissue was obtained from archived LN, TBM and MN samples surplus to clinical use and where adequate tissue was available for analysis. Lupus biopsies with either mixed lesions or significant scarring were excluded. TBM was used as a disease control due to lack of availability of normal kidney biopsies. Clinical parameters were collected from the health records retrospectively. The Interferon (IFN) score matrix was obtained as previously reported.^21^ Complete response was defined as a urine protein/creatinine ratio (uPCR) of <50 mg/mmol and an estimated glomerular filtration rate (eGFR) of ≥60 mL/min, or if <60 mL/min at baseline not fallen by >20% by 1 year postbiopsy. Partial response was defined as a uPCR <300 mg/mmol with a≥50% improvement from baseline and eGFR criteria the same as for complete response. Non-response was defined as failing to achieve partial response by 1 year.

### RNA extraction

Six 10 μm thick whole tissue sections were obtained by microtome from each FFPE block, following removal of two 4 μm sections. To prevent cross-contamination, the equipment was cleaned with RNase Away between samples and a fresh blade was used for each sample. Whole tissue RNA isolation was performed the same day using the RNeasy FFPE Kit (Qiagen, #73504) and RNA concentration was evaluated by NanoDrop ND1000 spectrophotometer (Thermo Fisher Scientific, Waltham, Massachusetts, USA; online supplemental data S1).

10.1136/lupus-2021-000615.supp6 Supplementary data

### Transcriptome analysis

Transcriptome analysis was performed using 100 ng of total RNA on the NanoString nCounter platform (NanoString nCounter FLEX Dx analysis system, NanoString Technologies, Seattle, Washington, USA) with a probe CodeSet comprising the human PanCancer immune profiling panel (730 immune related genes, 40 housekeeping genes, 6 positive control genes, 8 negative control genes) and additional 20 custom probes selected based on literature review (online supplemental table 1). Samples were run using two CodeSets which differed only by 10 unique probes, enabling us to analyse 750 endogenous transcripts across the 78 samples. CodeSet One: LN class III (n=6), class IV (n=9), class V (n=8), TBM (n=8), MN (n=1). CodeSet Two: LN class III (n=5), class IV (n=14), class V (n=13), TBM (n=6), MN (n=8). All samples passed NanoString quality control parameters (image quality control, binding density, positive control linearity and limit of detection). To control for inter-CodeSet variation and batch variability we used the nSolver Cross Reporter Library File (RLF) function to calibrate raw counts of overlapping probes using an identical sample run on both CodeSets. To reduce technical bias, and increase confidence and reproducibility, background thresholding was set at 100 counts, which is >double (mean+2 SD) of negative control probes. Counts were normalised to the geometric mean of 10 housekeeping genes (*FCF1, HPRT1, MRPS5, MTMR14, POLR2A, PRPF38A, SDHA, SF3A3, TUBB* and *ZC3H14*), selected as they were expressed across all sample types. The normalised counts for all samples are listed in online supplemental data S2.

10.1136/lupus-2021-000615.supp1 Supplementary data

### Statistical analysis

Differential gene expression (DGE) was performed using the nSolver advanced analysis (V.4) Fast/Approximate algorithm which uses the simplified negative binomial model for all probes except where the algorithm does not converge and the linear regression method is used. LN, MN or the LN class (III, IV and V) were used as the independent variables and TBM as our reference group. Threshold count was set at 100 and the observational frequency within samples set at 8%; P value was adjusted using Benjamini-Hochberg method with the false discovery threshold for set at 0.05. Volcano plots were produced using the Enhanced Volcano package (https://github.com/kevinblighe/EnhancedVolcano) in R.^22^ Functional protein-association network visualisation was performed using the STRING database (https://string-db.org/), using the full STRING network. Network edges were defined by confidence, and interactions were set to the highest confidence interaction score (≥0.9). Disconnected nodes within networks were not displayed. Significant differentially expressed genes were evaluated for enrichment of Gene Ontology (GO) terms (Biological Processes, Molecular Functions, Cellular Component), Reactome pathways and STRING local network clusters. Visual interaction networks were generated with functional enrichments in GO terms (Biological Processes) and STRING analysis. Area proportional Venn Diagram of overlapping differentially expressed genes between LN subclasses was generated with BioVenn (www.biovenn.nl).^23^ Data from advanced analysis nSolver software were read into the R statistical environment and data visualisation was performed using boxplots, principal components analysis, volcano plots and heatmaps. Heatmaps were generated using Z scores of normalised counts and pheatmap in R. Clusters were analysed using Fisher exact test. Analysis of variance with Sidak’s multiple comparisons test was used for comparison of multiple groups. Data were analysed using GraphPad Prism (V.8.0).

### Patient and public involvement (PPI) statement

This study was part of the MASTERPLANS consortium and patient collaborators participated in every work-strand including this study. A glossary of terms was produced by PPI to allow further engagement with the wider patient community and research findings disseminated at lay review days. Lay reviews of publications are shared on the MASTERPLANS website (https://sites.manchester.ac.uk/masterplans).

## Results

### Characterisation of the cohort

From our archived FFPE kidney biopsies, we identified 55 LN biopsies containing sufficient tissue for analysis. Biopsies were from female patients and consisted of class III (n=11), class IV (n=23) and class V (n=21) LN biopsies. Only Class III and IV LN biopsies with active lesions were included (ie, A or A/C but not C). We used TBM disease (n=14) as controls. We also compared class V LN with MN (n=9) given clear histological similarities on light microscopy but different aetiologies. Demographic, clinical and biopsy data are depicted in table 1. Biopsy ages ranged from under 1 year to over 11 years old. Active glomerular lesions, glomerular crescents and necrosis were present in the class III and IV but not the class V LN biopsies. Glomerular thrombosis was only identified in one class III biopsy. Tubuloreticular inclusions (TRIs) were identified in most class III (91%), class IV (78%) and class V (81%) biopsies. IFTA was low across all the classes. Serum creatinine and urine protein:creatinine ratio were higher in class IV compared with either class III or class V LN (table 1, online supplemental figure 1A and B). Serum albumin was reduced in all classes relative to TBM (table 1, online supplemental figure 1B). Complements C3 and C4 were significantly reduced in both class III and class IV in comparison to class V LN (table 1, online supplemental figure 1C).

10.1136/lupus-2021-000615.supp3 Supplementary data

**Table 1:** Patient demographics and clinical data

	Lupus nephritis class III (n=11)	Lupus nephritis class IV (n=23)	Lupus nephritis class V (n=21)	Thin basement membrane (n=14)	Membranous nephropathy (n=9)
Female/male (%F)	11/0 (100%)	23/0 (100%)	21/0 (100%)	13/1 (92.9%)	5/4 (55.6%)
Age of patient at time of kidney biopsy (years)	33 (16–58)	36 (22–48)	36 (19–57)	38.5 (16–67)	53 (42–73)
Disease duration at time of kidney biopsy (years)	0 (0–17)	8 (0–28)	5 (0–32)		
First kidney biopsy—number (%)	10 (91%)	13 (57%)	9 (43%)		
Ethnicity—number (%)					
Caucasian	2 (18%)	7 (30%)	3 (14%)	4 (28.6%)	3 (33.3%)
Black	5 (45%)	7 (30%)	11 (52%)	1 (7.1%)	0 (0%)
East Asian	2 (18%)	8 (35%)	5 (24%)	3 (21.4%)	4 (44.4%)
South Asian	0	0	1 (5%)	6 (42.9%)	2 (22.2%)
Other	2 (18%)	1 (4%)	1 (5%)		
Renal function at time of kidney biopsy:					
Serum albumin—g/L (NR 35–50)	32 (20–41)†	21 (12–35)‡	28 (13-40)§	37 (31–47)	31 (16–40)¶
Serum creatinine—micromol/l (NR 55–110)	70 (41–135)	95 (53–310)††	61 (45–146)	64 (55–179)	80 (56–180)
eGFR—mL/min/1.73 m^2^ (NR >89)	76 (50–90)	64 (14–90)‡‡	90 (32–90)	90 (25–90)	79 (24–90)
Urine protein:creatinine ratio—mg/mmol (NR <30)	82 (37–404)	666 (150–2414)§§	307 (0–1463)	10 (0–443)	552 (297–729)
Age of kidney biopsy at time of analysis (years)	3.55 (0.11–9.7)	4.7 (0.3–11.54)	4.34 (1.32–10.72)	3.1 (0.2–5)	1.2 (1–4.6)
Number of glomeruli	16 (9–29)	13 (8–30)	17 (7–37)	16.5 (7–32)	15 (6–26)
Number of active glomeruli	4 (1–7)	11 (1–25)	0 (0)	0	0
Number of sclerosed glomeruli	0 (0–4)	2 (0–10)	1 (0–9)	1.5 (0–5)	1 (0–8)
Number of glomeruli with crescents	0 (0–2)	2 (0–10)	0 (0)	0	0 (0–1)
Number of glomeruli with thrombosis	0 (0–1)	0 (0)	0 (0)	0	0
Number of glomeruli with necrosis	5 (45%)	1 (4.3%)	0 (0%)	0	0
Acute tubular injury or tubulitis—number (%)	5 (45%)	16 (70%)	9 (43%)	0	0
Tubuloreticular inclusions—number (%)	10 (91%)	18 (78%)	17 (81%)	0	1 (11.1%)
Interstitial fibrosis and tubular atrophy (%)	0 (0–20; n=3)	5 (0–40; n=13)	5 (0–25; n=11)	2.5 (0–10)	10 (0–50)
C3 g/L (NR 0.7–1.7)	0.52 (0.22–0.76)	0.6 (0.23–1.22)	1.09 (0.49–1.82)¶¶		
C4 g/L (NR 0.16–0.54)	0.06 (0–0.25)	0.09 (0.03–0.22)	0.19 (0.07–0.46)†††		
dsDNA IU/mL (NR 0–30)	2041 (248–20171)	275 (17–5789)‡‡‡	41 (0–723)§§§		
Treatment					
First biopsy (number)	10	13	9		
MMF and RTX	10 (100%)	11 (85%)	4 (44%)		
CYC and RTX	0	1 (8%)	1 (11%)		
MMF and HCQ	0	1 (8%)	1 (11%)		
Other	0	0.0	3 (33.3%)		
Previous biopsy (number)	1	10	12		
MMF and RTX	0	1 (10%)	3 (25%)		
CYC and RTX	0	1 (10%)	2 (17%)		
CYC	1 (100%)	6 (60%)	0		
RTX added to MMF	0	1 (10%)	7 (58%)		
MMF	0	0	0		
No Rx escalation	0	1 (10%)	0		
Response					
Complete/partial/non-response	9 (82%)/1 (9%)/1 (9%)	10 (43%)/0/13 (57%)	11 (52%)/3 (14%)/7 (33%)		

Values represent median (range of values) or number (%).

†* vs TBM.

‡**** vs TBM and ** vs class III.

§**** vs TBM.

¶** vs TBM.

††* vs class III and *** vs class IV.

‡‡** vs class IV.

§§*** vs class III and ** vs class V and **** vs TBM

¶¶**** vs class III or class IV.

†††*** vs class III or class IV.

‡‡‡* vs class III.

§§§** vs class III.

*P≤0.05, **p≤0.01, ***p≤0.001, ****p≤0.0001 derived from ANOVA with Sidak’s multiple comparisons test.

ANOVA, analysis of variance; CYC, cyclophosphamide; eGFR, estimated glomerular filtration rate; HCQ, hydroxychloroquine; MMF, mycophenolate mofetil; NR, normal range; RTX, rituximab; TBM, thin basement membrane.

### Immune gene expression analysis in LN renal biopsies

**Figure 1:**
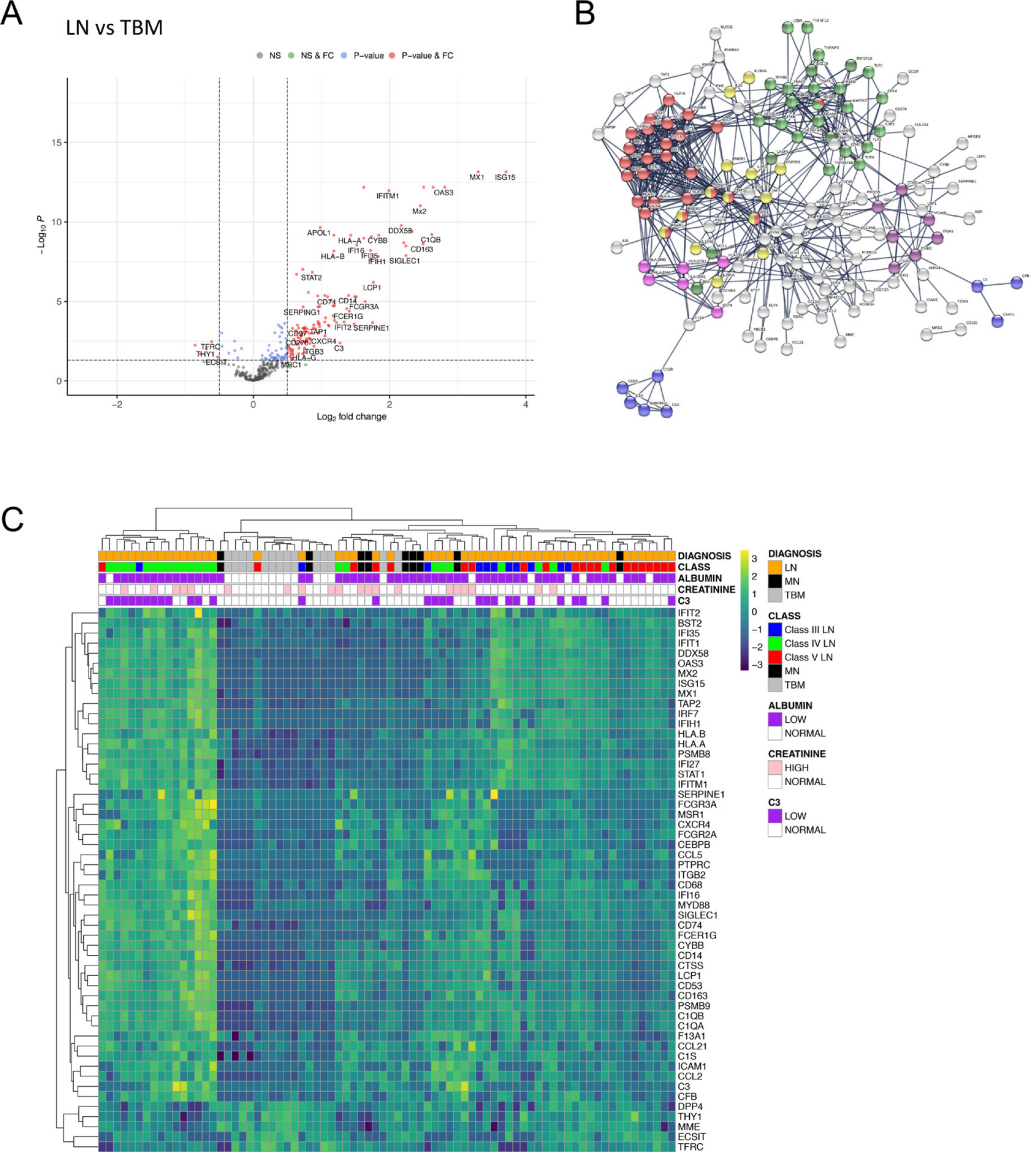
DGE in LN compared with TBM disease. (A) Volcano plot depicting DGE of LN (n=55 biopsies) vs TBM disease (n=14 biopsies). Benjamini-Hochberg adjusted p=0.05 and log2 FC cut-off=0.5. (B) STRING interaction network for all 171 significant differentially expressed genes between LN and TBM (highest confidence score=0.9). Red nodes—type I IFN; blue nodes—complement cascade; pink nodes—MHC II; yellow nodes—JAK-STAT signalling; purple nodes—integrin binding; light green nodes—NF-κB+TIR domain; dark green nodes—NF-κB and apoptosis modulation. (C) Heatmap of all significantly increased differentially expressed transcripts with log2 FC ≥1 (n=49) and all significantly downregulated transcripts with log2 FC ≤−0.5 (n=5) identified in LN vs TBM, across the entire cohort (LN n=55 biopsies, MN n=9 biopsies, TBM n=14 biopsies). DGE, differential gene expression; FC, fold change; LN, lupus nephritis; MN, membranous nephropathy; NS, non-significant; TBM, thin basement membrane.

We first compared gene expression between all LN classes and TBM samples and identified 171 (159 increased and 12 decreased) differentially expressed genes (figure 1A, online supplemental data S3). Pathway analyses showed that type I IFN signalling was highly enriched in LN over controls in addition to JAK-STAT signalling, complement cascade, MHC II, Integrin binding, NF-κB and apoptosis pathways (figure 1B, online supplemental data S4 and S5). Hierarchical clustering using transcripts with a log2 fold change (FC) of ≥1 (n=49) or ≤−0.5 (n=5) over the entire cohort revealed three main clusters (figure 1C). From left to right in figure 1C, group 1 contained only LN samples (n=16), group 2 contained all but one of the TBM samples (n=13 TBM, n=3 LN) and group 3 was LN-dominant (n=36 LN vs n=1 TBM). As reported,^16^ there was no clear relationship between molecular clusters and histological LN classes (figure 1C).

### Comparison of class V LN and idiopathic MN

**Figure 2:**
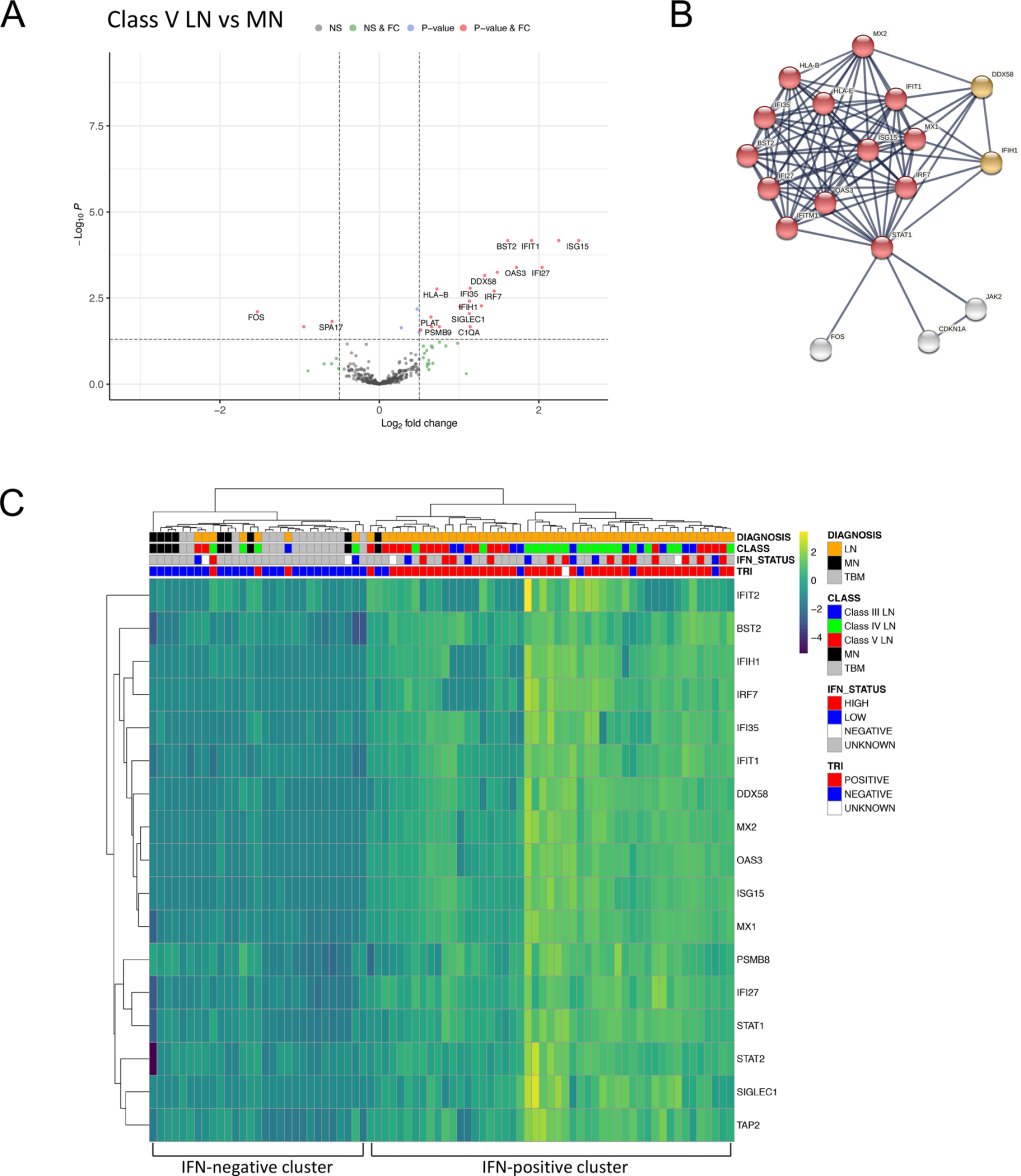
DGE in class V lupus nephritis compared with idiopathic MN. (A) Volcano plot depicting DGE of class V LN (n=21 biopsies) vs MN (n=9 biopsies). Benjamini-Hochberg adjusted p=0.05 and log2 FC cut-off=0.5. (B) STRING interaction network for all 26 significant differentially expressed genes between class V LN and MN (highest confidence score=0.9). Red nodes—type I IFN; orange nodes—regulation of type III IFN. (C) Heatmap of type I IFN-associated genes across the entire cohort (LN n=55 biopsies, MN n=9 biopsies, TBM n=14 biopsies). IFN status refers to blood IFN score (see methods). DGE, differential gene expression; FC, fold change; MN, membranous nephropathy; TRI, tubuloreticular inclusions.

The histological lesion in class V LN is a diffuse membranous glomerulopathy with similarities to idiopathic MN. When we compared class V LN with MN samples there were 26 (23 higher and 3 lower expression) differentially expressed genes (figure 2A, online supplemental data S6). The top enrichment term was type I IFN signalling (figure 2B, online supplemental data S4 and S7). A core set of 67 type I IFN-associated genes is upregulated across leucocyte subsets (neutrophils, CD4 and CD8 positive T cells, monocytes) in SLE.^24^ Our panel contained probes targeting 17 of these genes and 13 of the 23 transcripts with higher expression were part of this core set, indicating that the type I IFN signature is a key molecular distinction between class V LN and MN.

Hierarchical clustering using the 17 type I IFN-associated transcripts separated LN from both the MN and TBM samples (figure 2C). The proportion of LN biopsies in the IFN-positive cluster (n=48, 87%) was significantly greater than that in the IFN-negative cluster (n=7, 13%, p<0.0001, Fisher exact test). Within the IFN-positive cluster, the next hierarchy contained two clusters, with higher or lower IFN expression. The proportion of class V LN samples in the cluster with lower IFN expression (n=14, 74%) was significantly greater than that in the cluster with higher IFN expression (n=5, 26%, p=0.0009, Fisher exact test). TRIs in glomerular endothelial cells is frequent in LN and related to the type I IFN response. TRIs were present in 45 out of 54 LN biopsies (83%, data unavailable for one biopsy) and of the 47 biopsies in the IFN-positive cluster, 89% (n=42) had TRIs (figure 2C). We measured the blood type I IFN signature in 25 (46%) of the 55 patients with LN. A high blood IFN score was present in 13 (62%) of the 21 patients within the renal biopsy IFN-positive cluster and 1 of the 5 (20%) of patients within the renal biopsy IFN-negative cluster. There were no significant differentially expressed genes between MN and TBM samples (online supplemental figure 2, online supplemental data S15).

10.1136/lupus-2021-000615.supp4 Supplementary data

### Gene expression analysis within the histological LN classes

**Figure 3:**
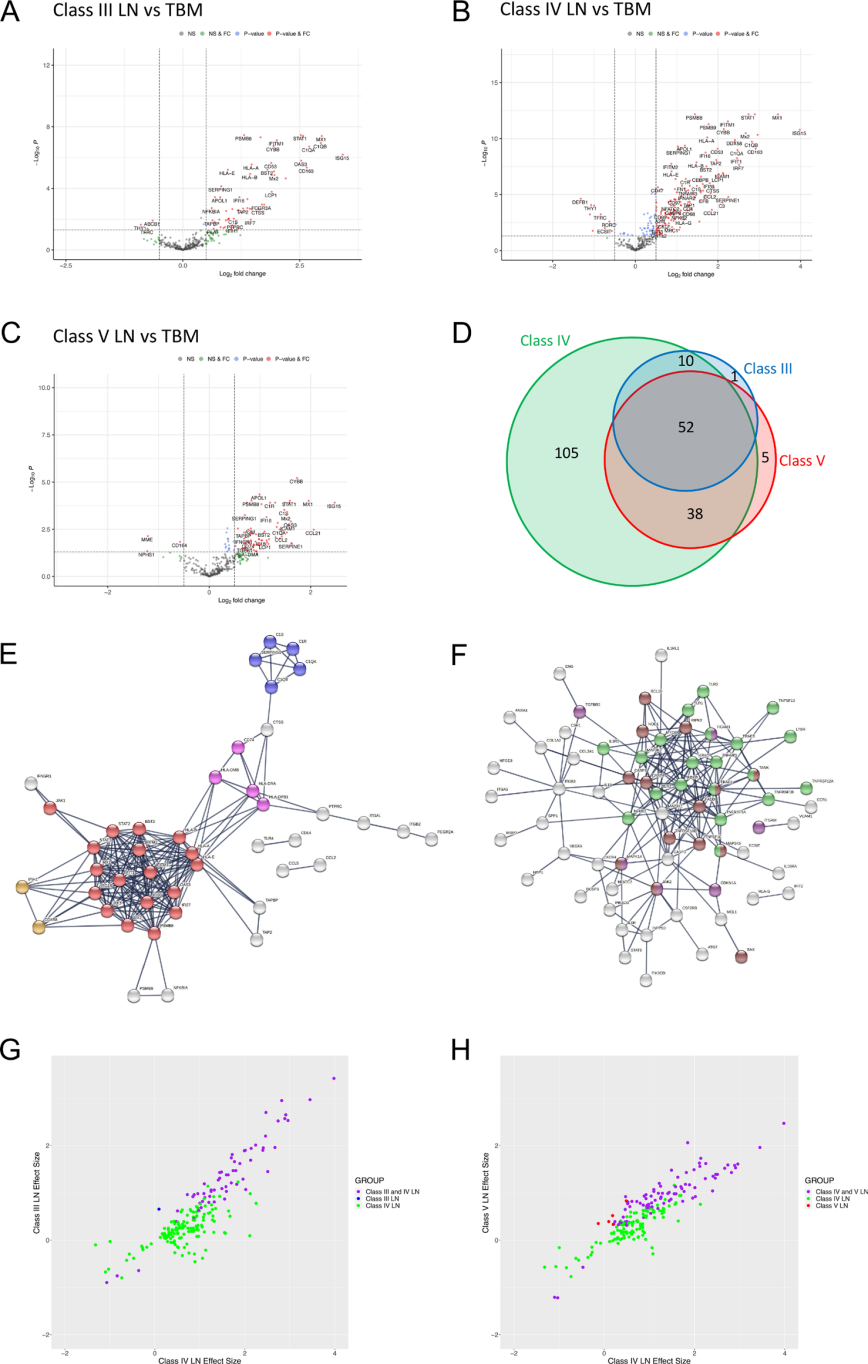
Immune gene expression analyses in LN subclasses. (A–C) Volcano plots depicting differentially expressed genes in (A) class III LN (n=11 biopsies), (B) class IV LN (n=23 biopsies) and (C) class V LN (n=21 biopsies) vs TBM disease (n=14 biopsies). Benjamini-Hochberg adjusted p=0.05 and log2 FC cut-off=0.5. (D) Area proportional Venn diagram of overlapping differentially expressed genes between class III (63 genes), class IV (205 genes) and class V (95 genes) LN. (E, F) STRING interaction networks using the highest confidence score (0.9) for all 52 significant differentially expressed genes that were common to all LN classes (E) and all 105 genes that were only significant in class IV LN (F). Red nodes—type I IFN; blue nodes—complement cascade; pink nodes—MHC II; orange nodes—regulation of type III IFN; light green nodes—NF-κB+TIR domain; purple nodes—positive regulation of ROS metabolic process; brown nodes—positive regulation of pepsidase activity. (G, H) Plots comparing the effect sizes of all 205 differentially expressed genes in class IV LN with either class III LN (G) or class V LN (H). Data points are coloured according to groups defined in the Venn diagram (D), with intersections shown in purple. FC, fold change; LN, lupus nephritis; TBM, thin basement membrane.

We next performed DGE (figure 3A–C) and pathway analysis (online supplemental figure 3 and online supplemental data S4, S16–S18) for each LN class. Compared with TBM disease, in class III LN there were 63 differentially expressed genes (60 increased and 3 decreased, figure 3A, online supplemental data S8); in class IV LN 205 differentially expressed genes (189 increased and 16 decreased, figure 3B, online supplemental data S9) and in class V LN 95 differentially expressed genes (92 increased and 3 decreased, figure 3C, online supplemental data S10). There were 52 significant differentially expressed genes common across classes (figure 3D, online supplemental table 2) and pathway analysis showed enrichment for type I IFN signalling, complement and MHC II pathways (figure 3E, online supplemental data S4 and S11). The FC was greater in class IV for all 52 genes compared with class V and for 85% of the genes compared with class III (online supplemental table 2).

10.1136/lupus-2021-000615.supp5 Supplementary data

10.1136/lupus-2021-000615.supp2 Supplementary data

Compared with TBM disease, there were 105 genes that were significantly differentially expressed in class IV but not in either class III or V LN biopsies (figure 3D). Pathway analysis showed enrichment for NF-κB signalling and Toll-IL1-resistance domain, positive regulation of reactive oxidative species (ROS) metabolic processes and positive regulation of pepsidase activity pathways (figure 3F, online supplemental data S4 and S12). When we plotted the effect sizes of all 205 significantly differentially expressed class IV transcripts with the respective effect sizes for class III (figure 3G) and class IV (figure 3H) LN. most of the changes were concordant. This indicated that only a small number of transcripts in our dataset were uniquely differentially regulated in class IV LN biopsies (ie, where significant effect sizes in class IV LN transcripts had low or zero effect sizes in either class III or class IV LN).

**Figure 4:**
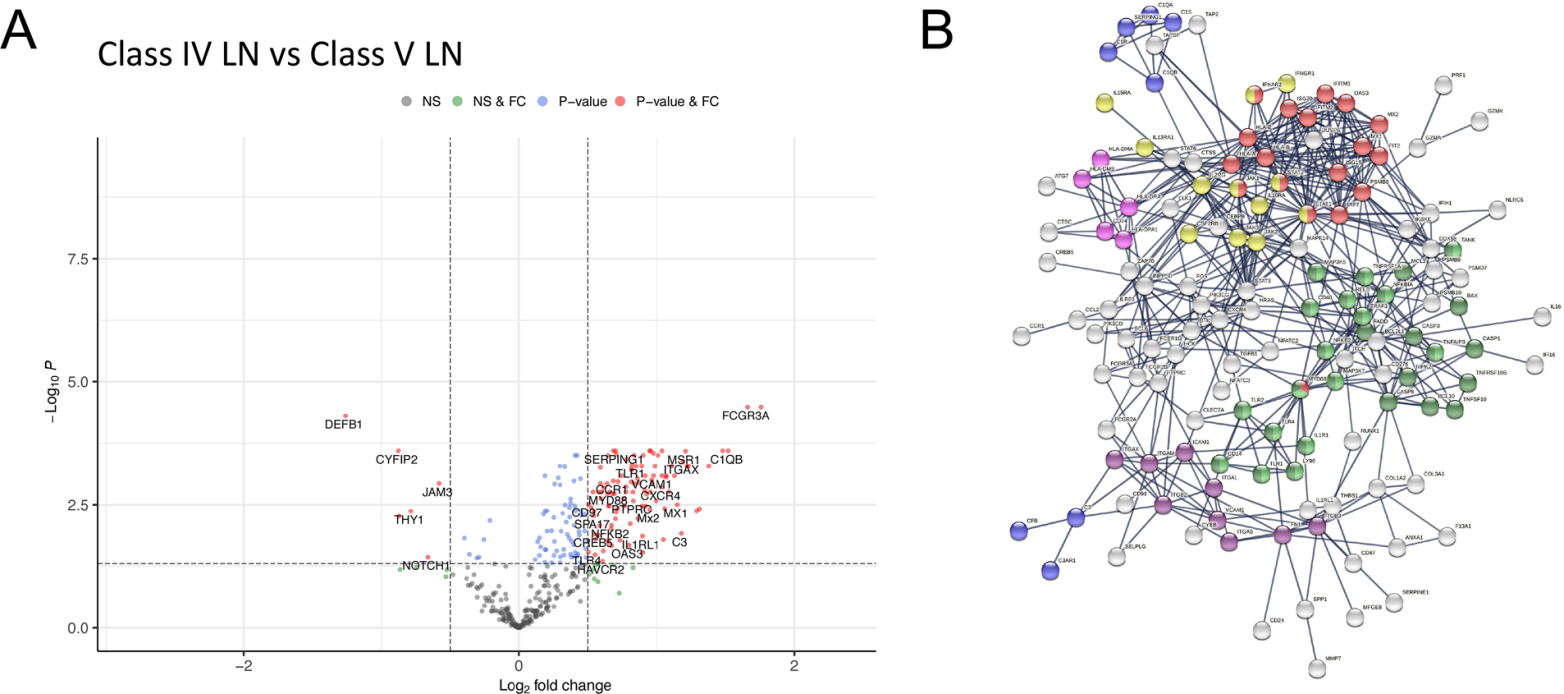
Differential gene expression between class IV and class V LN. Volcano plots depicting differential gene expression (DGE) of class IV (n=23 biopsies) vs class V (n=21 biopsies). Benjamini-Hochberg adjusted p=0.05 and log2 FC cut-off=0.5. (B) STRING interaction network for 167 significant differentially expressed genes with expression higher in class IV vs class V (highest confidence score=0.9). Red nodes—type I IFN; blue nodes—complement cascade; pink nodes—MHC II; yellow nodes—JAK-STAT signalling; purple nodes—integrin binding; light green nodes—NF-κB+TIR domain; dark green nodes—NF-κB and apoptosis modulation. FC, fold change; LN, lupus nephritis; NS, non-significant.

To explore differences between proliferative and membranous LN, we compared class IV with class V LN. This showed 179 (167 higher and 12 lower expression) differentially expressed genes in class IV (figure 4A, online supplemental data S13). Pathway analysis showed enrichment for type I IFN signalling, JAK-STAT signalling, Complement Cascade, MHC II, Integrin binding, NF-κB and apoptosis pathways (figure 4B, online supplemental data S4 and S14). While most of these pathways were also identified in our single subclass analysis of class IV and V compared with TBM disease (online supplemental figure 3BC), respectively, we observed an additional significant upregulation and enrichment for these pathways in class IV relative to class V (figure 4B).

## Discussion

Our study and others^16 18 19^ have demonstrated the feasibility of using NanoString technology to quantitate transcript expression on FFPE renal biopsy tissue and it is remarkable that this was possible using biopsies that were up to 11 years old. This approach has potential clinical utility since FFPE tissue is an integral component of the diagnostic renal biopsy pathway. In our comparison of LN with TBM disease, 36 significant STRING network clusters were identified, with the top 10 terms involving NF-κB (top 6 STRING clusters, 8 of 36 total) or IFN alpha/beta signalling (6 of 36 STRING clusters). Complement was also highly represented (6 of 36 STRING clusters). Consistent with published data, we could detect a type I IFN signature across all three LN classes.^16^ This IFN-associated transcriptomic signature was so well represented in LN that it clearly differentiated between class V LN and MN, despite their striking morphological similarities. Interestingly, there were no significantly increased transcripts when we compared MN to TBM.

The molecular profile of LN was first studied using laser-captured glomeruli from frozen tissue from patients with proliferative LN (class III and IV).^17^ Four gene clusters identifying B cells, a type I IFN response, myeloid lineage and the production of extracellular matrix were described. Notably, there was marked heterogeneity between biopsies (which could be for both technical and biological reasons) and no distinct profile between class III and IV biopsies. In a study that included proliferative, mixed and class V LN and which analysed the glomerular and tubulointerstitial compartments separately, the abundance of RNA transcripts did not correlate with either histological class or the National Institute of Health activity and chronicity indices.^16^ Similarly, in a study of 28 paired biopsies from 14 patients with LN, assessed using a NanoString immune panel analogous to our study, there were no distinct clusters when RNA transcript abundance was analysed from either glomerular or tubulointerstitial compartments.^18^ A major strength of our study was the selection of well-defined class III, IV and V biopsies with no mixed lesions. Despite this approach, and in agreement with the studies outlined above, we could not detect a distinct transcriptomic signature for each histological subtype.

We did, however, identify 52 differentially expressed genes in all three LN classes. STRING analysis demonstrated enrichment for IFN signalling, MHC II and classical antibody-mediated complement activation. The FC in class IV LN was greater for most of these genes in comparison to either class III or V. It is notable that the intrarenal expression of IFN-associated genes is influenced by disease flare and treatment, with *MX1*, *STAT1* and *IRF7* all upregulated at flare, and reduced or further elevated in the post-treatment biopsy of treatment or non-responders, respectively.^19^ In this and our analysis, it was not possible to determine the contributions of the IFN-response from resident glomerular endothelial cells and from infiltrating leucocytes, the signal from the latter more likely to be influenced by anti-inflammatory therapy.

We identified 105 significantly differentially expressed genes in class IV LN where all STRING enrichment clusters identified were involved in NF-κB and apoptosis. Accumulating evidence implicates NF-κB in the pathogenesis of LN including podocyte injury,^25^ and NF-κB-mediated cytokine expression has been highlighted in non-response.^19^ Variants of several genes in TLR/ NF-κB signalling are associated with LN, including *TLR 3/7/9, MYD88, IRAK1, TNFAIP3* and *TNIP1*,^26^ but this is the first time that NF-κB has been attributed to class IV LN. VCAM-1 has been suggested as an LN activity biomarker,^27^ with increased expression between initial and flare biopsy^18^ and the ability to differentiate class IV from other classes. In agreement, we only observed significant elevation in our study in class IV LN. Our class IV analysis also highlighted regulation of ROS metabolic processes, which has been reported as having a role in SLE.^21^ *CYBB*, the gene that encodes the beta chain of cytochrome-245, a subunit of the NADPH oxidase enzyme complex, and the M2 macrophage marker *CD163* were increased in all classes but most marked in class IV. CD163 has been suggested as a urinary biomarker of activity in LN.^18^ The presence of type I IFN signalling has been associated with mitochondrial abnormalities, leading to mitochondrial insufficiency and increased cell death as a regulatory mechanism in persistent type I IFN response.^21^ However, it has been demonstrated in animal models that altered metabolic dysfunction is a reversible change in lupus affected tissues, not driven by type I IFN exposure, and correct modulation can be restored after immunosuppression in animal models.^28^

We observed a similar STRING interaction network in our class IV vs class V and our class IV versus TBM analysis. In fact, comparing the two, 71.4% (135 of 189) of the genes identified relative to TBM are also significantly higher when comparing class IV to class V, with pathways enriched for type I IFN signalling, JAK-STAT signalling, Complement Cascade, MHC II, Integrin binding, NF-κB and apoptosis. From this, we can conclude that the immunological enrichment in class IV LN is significantly upregulated from our disease control (the TBM biopsies) and from class V LN.

Exploratory analysis identified osteopontin (*OPN/SPP1*), fibronectin (*FN1*) and galectin-3 (*LGALS3*) as genes of interest with respect to lupus disease activity.^16^ *FN1* has also been linked to flare.^18^ In our study, *LGALS3* was not significantly expressed. However, *FN1* was significant in both class IV and V. *OPN* was significant in class IV only, an observation not drawn in the original study. *OPN* was proposed as a candidate marker of aggressive and proliferative LN^16^ and has been associated with hypercellularity, cellular proliferation and crescent formation in murine LN.^29^

A key goal of our study was to provide whole kidney transcriptomic profiles for class III, IV and V LN with the aim of identifying molecular pathways and inflammatory molecules that could be targeted for therapy. While we did not identify new pathways for targeting in LN, the recent approval of anifrolumab, a human monoclonal blocking antibody to the type I IFN receptor subunit 1, for lupus treatment provides an opportunity to understand how the intrarenal IFN signature might influence response. Complement inhibition may also be a potential therapy in LN and using renal complement markers, such as combining complement immunostaining and transcriptomic profiles, may be a method of selecting patients for proof of concept studies.^30^

Our study has important limitations. The transcriptomic analysis was limited to the 750 transcripts interrogated by our NanoString panel. However, the panel was selected for its ability to interrogate immune and inflammation-related transcripts which are clearly of major importance in understanding the molecular pathogenesis of LN. We used whole renal tissue sections rather than separating glomerular and tubulointerstitial tissue, so we cannot distinguish expression from different renal compartments. Using whole tissue has the advantage of being the most technically feasible approach experimentally and most practical for downstream clinical application. This was a retrospective study and so the treatment approaches were heterogeneous. We limited our biopsies to well-defined class III, IV and V lesions without significant scarring to strengthen our ability to detect differences between the classes. How the signals would be affected by mixed lesions or lesions with chronic damage (eg, arteriosclerosis, IFTA and glomerulosclerosis) would need further study.

In summary, we have provided a whole kidney section transcriptomic analysis of class III, IV and V LN biopsies using a panel of immune and inflammation gene probes. We identified pathways common to all three classes and characterised pathways that were only significantly modulated in our class IV biopsies. As more targeted treatments are developed for LN, it may be possible to use transcriptomic analysis to inform treatment selection.

## Data Availability

Data are available on reasonable request. All data relevant to the study are included in the article or uploaded as supplementary information. Data are provided in the supplemental excel file. Raw data files are available on request.
