# Supplementary material for: Immune gene expression and functional networks in distinct lupus nephritis classes
Source: Lupus Sci Med. 2022 Jan 24;9(1):e000615. doi: 10.1136/lupus-2021-000615 (PMC8788334; doi:10.1136/lupus-2021-000615)
Supplement: Supplementary data [file lupus-2021-000615supp001.pdf]

Gene	Accession #	Target Sequence (5'-3')
APOL1	NM_003661.3	CCGCTTTGACCGGGATTACCAGCAGTACCATGGACTACGGAAAGAAGTGGTGGACACAAGCCCAAGCCCACGACCTGGTCATCAAAAGCCTTGACAAATT
CD2AP	NM_012120.2	CTCATGAAGCCCAGGACGATTCAGAAACTGTTTTGGCTGGGCCTACTTCACCTATACCTTCTCTGGGAAATGTGAGTGAAACTGCATCTGGATCAGTTAC
Col1a2	NM_000089.3	CCAATGGATTTGCTGGTCCTGCTGGTGCTGCTGGTCAACCTGGTGCTAAAGGAGAAAGAGGAGCCAAAGGGCCTAAGGGTGAAAACGGTGTTGTTGGTCC
CTSB	NM_147780.2	ACAAAAACGGCCCCGTGGAGGGAGCTTTCTCTGTGTATTCGGACTTCCTGCTCTACAAGTCAGGAGTGTACCAACACGTCACCGGAGAGATGATGGGTGG
CTSC	NM_001114173.1	TGCTCGGTTATGGGACCACAAGAAAAAAAAGTAGTGGTGTACCTTCAGAAGCTGGATACAGCATATGATGACCTTGGCAATTCTGGCCATTTCACCATCA
DKK1	NM_012242.2	CGGCACGGTTTCGTGGGGACCCAGGCTTGCAAAGTGACGGTCATTTTCTCTTTCTTTCTCCCTCTTGAGTCCTTCTGAGATGATGGCTCTGGGCGCAGCG
IFI30	NM_006332.3	CAGGAACAAAATGTCAGTGGCAGGTGGGAGTTCAAGTGCCAGCATGGAGAAGAGGAGTGCAAATTCAACAAGGTGGAGGCCTGCGTGTTGGATGAACTTG
Kat2B	NM_003884.3	CATCTGGAGGCACCATCTCAACGAAGACTGCGATCTCCCAATGATGATATTTCTGGATACAAAGAGAACTACACAAGGTGGCTGTGTTACTGCAACGTGC
KLF2	NM_016270.2	GGAAGTTTGCGCGCTCAGACGAGCTCACGCGCCACTACCGAAAGCACACGGGCCACCGGCCATTCCAGTGCCATCTGTGCGATCGTGCCTTCTCGCGCTC
KLF4	NM_004235.4	CGAGCATTTTCCAGGTCGGACCACCTCGCCTTACACATGAAGAGGCATTTTTAAATCCCAGACAGTGGATATGACCCACACTGCCAGAAGAGAATTCAGT
MCL1	NM_021960.3	GCTGTAACTTCCTAGAGTTGCACCCTAGCAACCTAGCCAGAAAAGCAAGTGGCAAGAGGATTATGGCTAACAAGAATAAATACATGGGAAGAGTGCTCCC
MMP7	NM_002423.3	GTGCCAGATGTTGCAGAATACTCACTATTTCCAAATAGCCCAAAATGGACTTCCAAAGTGGTCACCTACAGGATCGTATCATATACTCGAGACTTACCGC
Mx2	NM_002463.1	AATTTACCGGCTCACTCAGGCGCGACACGCACTCTGTCAATTCTCCAGCAAAGAGATCCACTGAAGGGCGGCGATGCCTGTGGTTGTTTTCTTGTGCGTA
NPHS1	NM_004646.3	CCAGAGTAGAAACAAGGTGCATCCTGGGGTTGGCTTTAGAAACTAAACTTCTCCAAAAGGACAGGGCAGATTGTAAACGTCGTCTCAAAAATGAAATGCT
NPHS2	NM_014625.2	CCTTCCAAACCTGTTGAGCCACTAAATCCTAAAAAGAAAGACTCTCCCATGTTATAGGAAGGATGGGGCATAATGTGACTGTAAAGGGGCCTGCCATAGA
PLA1A	NM_015900.2	CAACCGAGTTTGGAAAAAAGACCGGACTACCATTATTGGGAAGTTCTGCACTGCCCTTTTGCCTGTCAATGACAGAGAAAAGATGGTCTGCTTACCTGAA
PLAT	NM_000931.2	CAAGGTTACCAACTACCTAGACTGGATTCGTGACAACATGCGACCGTGACCAGGAACACCCGACTCCTCAAAAGCAAATGAGATCCCGCCTCTTCTTCTT
SERPINE1	NM_000602.4	CGGAGCACGGTCAAGCAAGTGGACTTTTCAGAGGTGGAGAGAGCCAGATTCATCATCAATGACTGGGTGAAGACACACACAAAAGGTATGATCAGCAACT
TAGAP	NM_152133.2	TGAACAGGATGGACTCCAGCAATCTGGCCATCTGCATTGGACCCAACATGCTCACCCTGGAGAATGACCAGAGCCTGTCATTTGAAGCCCAGAAGGACCT
TGFBR2	NM_001024847.1	ATTTGGAGAATGTTGAGTCCTTCAAGCAGACCGATGTCTACTCCATGGCTCTGGTGCTCTGGGAAATGACATCTCGCTGTAATGCAGTGGGAGAAGTAAA

Supplemental Table 1: Custom Probes used across both CodeSets
