# Supplementary material for: Immune gene expression and functional networks in distinct lupus nephritis classes
Source: Lupus Sci Med. 2022 Jan 24;9(1):e000615. doi: 10.1136/lupus-2021-000615 (PMC8788334; doi:10.1136/lupus-2021-000615)
Supplement: Supplementary data [file lupus-2021-000615supp003.pdf]

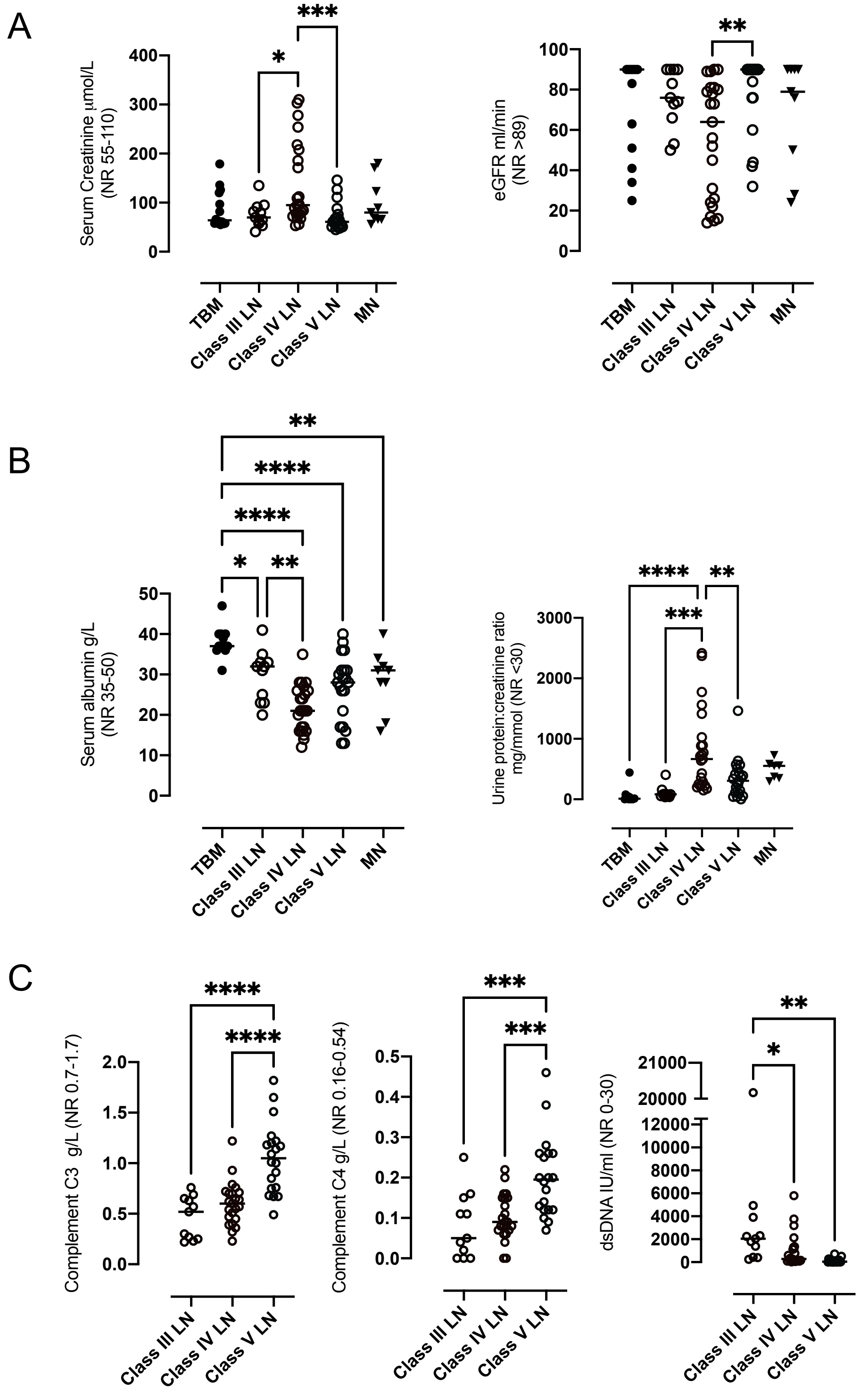

Supplemental Figure 1. **Renal function, complement and anti-double stranded DNA (dsDNA) antibody data at time of kidney biopsy.** (A) Serum creatinine and estimated glomerular filtration rate (eGFR), (B) serum albumin and urine protein:creatinine ratio (uPCR), and (C) Complement C3, C4 and dsDNA. Thin basement membrane (TBM) disease (black circles, n=14 patients); Lupus nephritis (LN) samples (open circles) categorized into class III (n=11 patients), class IV (n=23 patients) and class V (n=21 patients) nephritis; and membranous nephropathy (MN, black triangles, n=9 patients). Horizontal bars denote median values. ∗P ≤ 0.05, ∗∗P ≤ 0.01, ∗∗∗P ≤ 0.001, ∗∗∗∗P ≤ 0.0001. P values derived from one-way analysis of variance with Sidek multiple comparisons test. Missing values: albumin and uPCR data not available in n=1 TBM patient; uPCR data not available in n=2 MN patients; C3, C4 and dsDNA data not available in n=1 LN5 patient; and eGFR not available in n=1 TBM patient.
