# Supplementary material for: Immune gene expression and functional networks in distinct lupus nephritis classes
Source: Lupus Sci Med. 2022 Jan 24;9(1):e000615. doi: 10.1136/lupus-2021-000615 (PMC8788334; doi:10.1136/lupus-2021-000615)
Supplement: Supplementary data [file lupus-2021-000615supp004.pdf]

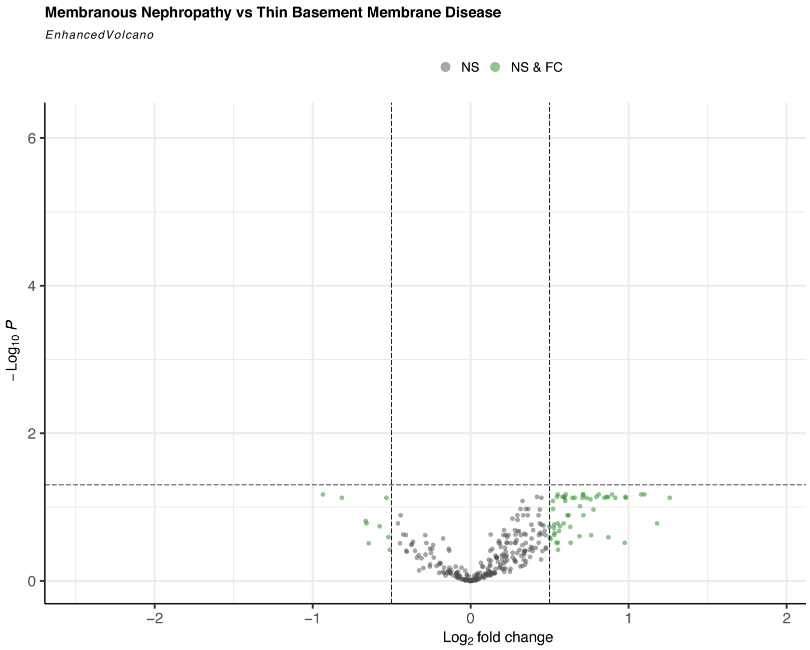


Supplemental Figure 2. **Differential Gene Expression analysis in Membranous Nephropathy (MN) versus Thin Basement Membrane (TBM) disease.** Volcano plot depicting differential gene expression of MN (n=9 biopsies) versus TBM (n=14 biopsies). Benjamini-Hochberg adjusted P value = 0.05 and log2 fold change (FC) cut-off = 0.5.
