# Supplementary material for: Immune gene expression and functional networks in distinct lupus nephritis classes
Source: Lupus Sci Med. 2022 Jan 24;9(1):e000615. doi: 10.1136/lupus-2021-000615 (PMC8788334; doi:10.1136/lupus-2021-000615)
Supplement: Supplementary data [file lupus-2021-000615supp005.pdf]

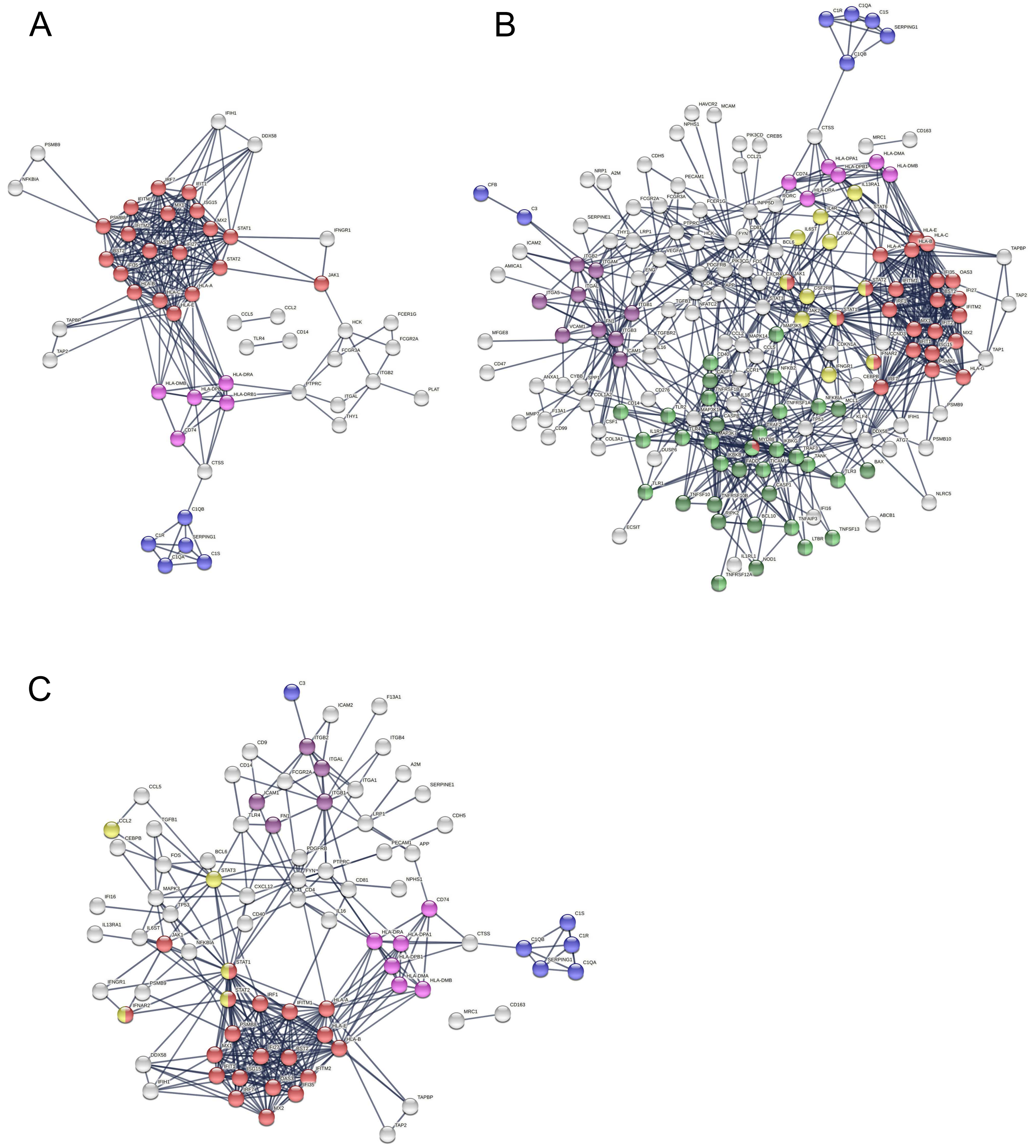

Supplemental Figure 3. **STRING interaction networks using all significant differentially expressed genes between LN classes and TBM disease.** (A) LN Class III (n=11 biopsies, 63 genes), (B) LN Class IV (n=23 biopsies, 205 genes), and (C) LN Class V (n=21 biopsies, 95 genes). Red nodes – Type I IFN; blue nodes – complement cascade; pink nodes – MHC II; yellow nodes - JAK-STAT signalling; purple nodes – Integrin binding; light green nodes – NFkB + TIR domain; dark green nodes – NFkB and apoptosis modulation. STRING analysis used the highest confidence score (0.9).
