# Supplementary material for: Immune gene expression and functional networks in distinct lupus nephritis classes
Source: Lupus Sci Med. 2022 Jan 24;9(1):e000615. doi: 10.1136/lupus-2021-000615 (PMC8788334; doi:10.1136/lupus-2021-000615)
Supplement: Supplementary data [file lupus-2021-000615supp002.pdf]

Gene Name	Class III		Class IV			Class V		
	FC	BH p value	Percent change	FC	BH p value	Percent change	FC	BH p value
**ISG15**	3.42	6.38E-07	16.37	3.98	1.64E-11	-27.78	2.47	0.000124
**MX1**	2.97	3.99E-08	16.16	3.45	6.79E-13	-34.01	1.96	9.79E-05
**C1QB**	2.95	1.14E-07	-4.41	2.82	1.88E-10	-51.53	1.43	0.00391
**C1QA**	2.70	1.94E-07	-8.52	2.47	1.06E-09	-48.52	1.39	0.00325
**CD163**	2.65	4.47E-06	9.81	2.91	7.90E-10	-42.26	1.53	0.00471
**IFI27**	2.57	3.81E-08	12.45	2.89	6.79E-13	-38.52	1.58	0.000124
**OAS3**	2.53	1.61E-06	17.00	2.96	4.60E-11	-36.36	1.61	0.00124
**STAT1**	2.52	3.49E-08	8.73	2.74	6.79E-13	-36.90	1.59	9.79E-05
**IFIT1**	2.20	2.24E-05	11.82	2.46	5.54E-09	-40.45	1.31	0.00843
**IFITM1**	2.01	7.45E-08	11.44	2.24	2.89E-12	-35.32	1.30	0.000124
**MX2**	1.96	1.38E-05	36.22	2.67	3.29E-11	-22.45	1.52	0.00056
**DDX58**	1.95	7.78E-06	23.59	2.41	1.32E-10	-30.77	1.35	0.00144
**CYBB**	1.94	1.76E-07	10.31	2.14	1.41E-11	-10.82	1.73	6.08E-06
**SIGLEC1**	1.90	1.38E-05	26.32	2.40	1.88E-10	-45.26	1.04	0.0122
**LCP1**	1.90	0.000168	5.79	2.01	2.47E-07	-41.58	1.11	0.0187
**CD53**	1.89	2.26E-06	5.29	1.99	7.90E-10	-21.16	1.49	0.000124
**BST2**	1.81	6.01E-06	-4.97	1.72	3.03E-08	-39.78	1.09	0.00415
**PSMB9**	1.66	4.87E-08	6.63	1.77	5.18E-12	-37.95	1.03	0.000148
**CTSS**	1.62	0.00227	16.67	1.89	2.29E-06	-27.78	1.17	0.0191
**IFI35**	1.47	0.00227	22.45	1.80	9.42E-07	-19.73	1.18	0.0106
**HLA-A**	1.47	2.82E-06	18.37	1.74	7.82E-11	-44.08	0.82	0.00487
**IRF7**	1.45	0.0106	73.10	2.51	2.24E-08	-4.14	1.39	0.00983
**HLA-B**	1.44	1.15E-05	2.78	1.48	1.34E-08	-39.86	0.87	0.00544
**ITGB2**	1.43	0.00209	0.70	1.44	1.84E-05	-29.37	1.01	0.0201
**CEBPB**	1.38	0.000498	15.22	1.59	2.47E-07	-28.99	0.98	0.0106
**CD14**	1.38	0.0019	18.12	1.63	1.15E-06	-3.62	1.33	0.00239
**PSMB8**	1.31	3.49E-08	9.92	1.44	6.79E-13	-36.49	0.83	9.79E-05
**TAP2**	1.29	0.00206	51.94	1.96	8.22E-09	-31.09	0.89	0.0223
**FCGR2A**	1.27	0.00344	11.02	1.41	9.80E-06	-22.36	0.99	0.017
**IFI16**	1.21	0.000378	40.50	1.70	1.79E-09	-6.61	1.13	0.000715
**HLA-DMB**	1.19	0.00254	-7.56	1.10	8.83E-05	-10.08	1.07	0.00517
**CCL2**	1.18	0.0412	54.24	1.82	7.72E-06	21.19	1.43	0.00768
**PTPRC**	1.13	0.0198	39.82	1.58	6.39E-06	0.00	1.13	0.0122
**C1S**	1.09	0.00919	36.70	1.49	1.67E-06	35.78	1.48	0.000297
**IFIH1**	1.07	0.0348	88.79	2.02	1.48E-07	-13.93	0.92	0.0457
**TLR4**	1.06	0.0248	17.92	1.25	0.000108	-16.89	0.88	0.0408
**CD74**	1.06	0.00221	24.53	1.32	5.79E-07	-26.51	0.78	0.0167
**CCL5**	1.03	0.0473	21.36	1.25	0.000269	10.68	1.14	0.0155
**ITGAL**	0.98	0.00926	-2.85	0.96	0.000254	-26.25	0.73	0.039
**HLA-DPB1**	0.96	0.0106	-25.03	0.72	0.00388	-15.16	0.82	0.0193
**HLA-E**	0.96	6.13E-06	-9.15	0.87	8.33E-08	-50.00	0.48	0.0143
**C1R**	0.93	0.00292	31.75	1.22	4.04E-07	29.59	1.20	0.000124
**F13A1**	0.87	0.0375	78.37	1.55	4.94E-07	12.89	0.98	0.0106
**SERPING1**	0.82	7.28E-05	29.27	1.06	9.22E-10	-14.15	0.70	0.000558
**APOL1**	0.80	0.000378	50.38	1.20	4.23E-10	24.19	0.99	4.52E-05
**HLA-DRA**	0.78	0.0106	28.21	1.00	6.39E-06	-11.28	0.69	0.0155
**STAT2**	0.69	0.000378	49.93	1.03	5.01E-10	-17.47	0.57	0.0029
**IFNGR1**	0.68	0.0151	40.53	0.96	3.31E-06	1.17	0.69	0.00983
**TAPBP**	0.63	0.0119	53.55	0.97	4.04E-07	8.37	0.69	0.00471
**NFKBIA**	0.62	0.00191	-18.61	0.50	0.00026	-36.41	0.39	0.034
**IFITM2**	0.45	0.0214	92.44	0.87	1.79E-08	64.67	0.74	0.000124
**JAK1**	0.39	0.00824	21.12	0.48	9.17E-06	-7.38	0.36	0.00988

Supplemental Table 2: 52 Genes statistically significant in all lupus nephritis (LN) classes. Fold change (FC) and Benjamini-Hochberg (BH) adjusted p values obtained for each LN class relative to thin basement membrane disease samples. Genes are ordered from highest to lowest FC based on values obtained from Class III LN. Percentage change (%) from Class III FC is shown for Class IV and V LN.
